# Recovery of Proteolytic and Collagenolytic Activities from Viscera By-products of Rayfish (*Raja clavata*)

**DOI:** 10.3390/md7040803

**Published:** 2009-12-15

**Authors:** Miguel Anxo Murado, María del Pilar González, José Antonio Vázquez

**Affiliations:** Grupo de Reciclado y Valorización de Materiales Residuales, Instituto de Investigacións Mariñas (CSIC), r/Eduardo Cabello, 6. Vigo-36208, Galicia, Spain; E-Mails: recicla@iim.csic.es (M.A.M.); pgonzalez@iim.csic.es (M.d.P.G.)

**Keywords:** proteolytic and collagenolytic activity, by-products upgrading, rayfish viscera wastes, pH and temperature effects, mathematical modelling

## Abstract

The aim of this work was to study the recovery of proteolytic and collagenolytic activities from rayfish (*Raja clavata*) viscera wastes. Initially, different parts of the gastrointestinal tract by-products (stomach, duodenum section including pancreas, final intestine) were evaluated. The extracts from proximal intestine yielded the highest values of both enzymatic activities. Optimal conditions for protease activity quantification were established at pH = 6, T = 40 °C and incubation time ≤20 min. The mathematical equation used to model the joint effect of pH and temperature led to maximum activity at pH = 8.66 and 59.4 °C, respectively. Overcooled acetone was found to be best option for recovery of enzymatic activities in comparison with ethanol, PEG-4000, ammonium sulphate and ultrafiltration system. Finally, a simple and systematic protocol of partial purification and total recovery of proteases and collagenases was defined.

## 1. Introduction

Proteases (EC 3.4.21–24 and 99) are enzymes that catalyze the hydrolysis of proteins. Among these, collagenases are a specific group able to break the peptide bonds in collagen molecules, an essential protein of epithelial, cartilaginous and bony tissues. Both enzymes are products of great interest in biotechnology processes, textile and detergent sectors, medical and basic research as well as the leather, food and pharmaceutical industries [1–3]. Specially, they have a growing use in the processing of marine foods and treatment of marine wastes as for example: the production of protein hydrolysates, silages and fish sauce, peeled and squid softening, hydrolysis of marine wastes and water recovery, besides increasing yield of fish meal and foodstuff processing [4–8]. In most of these applications the market value of the final products is low for what the cost of production cannot be high [9,10]. Moreover, in several cases, a combination of proteolytic and collagenolytic activities (PRC) are required to obtain a total protein hydrolysis [6,11]. Therefore, an exhaustive purification of enzymes for these applications is not required and only a partial low cost purification would make its use acceptable as substitute of the commercial ones.

Nowadays, their common sources come from vegetables, terrestrial animals and, mainly, micro-organisms [12–19]. The low-use of those obtained from fish is partly due to the seasonal variations of the materials and its not very suitable smells for industrial applications far from the marine field, but it is basically due to the relative shortage of studies and specific developments, only recently initiated in a systematic way [20–24].

However, there is agreement that proteases from fish contribute diversity to the catalytic characteristics of the terrestrial ones as activity and/or stability over wider domains of pH and temperature besides certain substrate specificities [25–28]. The animal proteases and, mainly, the collagenases carry out an important role related with the morphogenesis but the highest proportions of these enzymes are associated to digestive functions. In cartilaginous fish the pancreas is one of the key organs for obtaining them, whereas in bony fish without pancreas the highest proteolytic activity is accumulated in the corresponding digestive section, that is, the pyloric ceca which are located towards the end of the stomach.

On the other hand, in coastline areas such as the Port of Vigo (Galicia, NW Spain) the large volume of fish wastes derived from the fishing activity produce contamination problems that require great management, recycling and valorization efforts [29–32]. In this sense, rayfish catch generates, in the Bay of Vigo, more than 3.5 tons per day of by-products with 25% of viscera residue.

The present work reported a simple protocol to obtain enzymatic preparations with PRC from viscera wastes of rayfish. In addition, a preliminary characterization of activity distribution along rayfish intestine and mathematical modelling of combined effects of pH and temperature was also carried out. Further studies of exhaustive characterizing type, properties and functions of PRC should be done.

## 2. Results and Discussion

### 2.1. Study of proteolytic activities from different gastrointestinal sections

**Figure 1.**
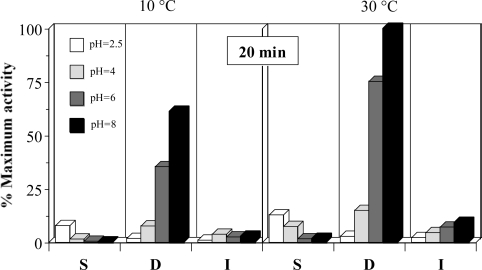
Proteolytic activities as % of maximum activity (pH 8, 30 °C and 20 minutes) obtained in different sections of viscera. S: stomach, D: duodenum, I: terminal intestine. The corresponding confidence intervals of independent experiments are not shown (α = 0.05, n = 3) because they are below 10% of the experimental mean value.

A preliminary study on the description of the PRC activities from the different gastrointestinal sections of rayfish was carried out. Figure 1 shows the results for the experimental conditions tested. These data indicate that the highest and significant activities are obtained, in all cases, in the duodenum section of rayfish viscera (*P* < 0.05). Moreover, the activity in D and I increases correlatively with the pH. In S section, the results are the opposite but the activity values are so low that this region lacked interest for later studies. Similar results were obtained when collagenolytic activities were measured (data not shown).

At the optimum pH, the activity in D falls about 40% when the temperature descends from 30 to 40 °C. The apparent activity per time unit is significantly lower at 40 min than 20 min (*P* < 0.05, data not shown). This can be due to the enzyme deactivation during the reaction time or, more likely, lack of linearity in the response at long times for exhaustion of the substrate (that could already happen at 20 min). Defining 1 EU of proteolytic activity as the enzyme concentration that generate 1 μM (181.2 μg) of tyrosine per min at 30 °C and pH 8, using casein as standard, and taking into account the ponderal proportions in the homogenates it can be established that D section has 8 EU per g of dry weight.

### 2.2. Optimization of the experimental conditions for proteolytic activity quantification

**Figure 2.**
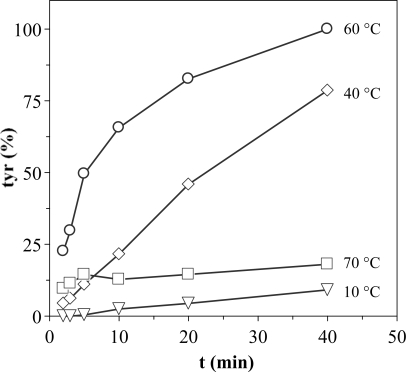
Production of tyrosine with the enzymatic reaction progress at pH 6 and four temperatures. Response as % of the maximum activity obtained at 60 °C and 40 min. The corresponding confidence intervals of independent experiments are not shown (α = 0.05, n = 3) because they are below 10% of the experimental mean value.

In order to improve the experimental conditions to measure proteolytic activities, a study of optimization of the enzymatic reaction progress was carried out. To establish the optimum test conditions firstly the reaction time during which tyrosine production maintains a reasonably constant slope with constant concentration of reagents should be known. Figure 2 shows the kinetics of tyrosine production using extracts from D section (*Procolax*) at pH 6 and different incubation temperatures. The activity increases from 10 to 40 °C without loss of the linearity throughout the time. The lack of linearity at 60 °C should be attributed to thermal deactivation and not to substrate deficit, since tyrosine levels outside of the linearity at 60 °C are lower than others inside the linearity at 40 °C. The thermal deactivation is very high at 70 °C.

Therefore, the appropriate conditions to apply the method of the casein are: pH 6, T = 40 °C, incubation time ≤20 min and the necessary dilution of the enzymatic sample to obtain a concentration of tyrosine in the range of 10–100 μg/mL. Under such conditions, the tyrosine production increases lineally with the progress of incubation and it is suitable to transform its value in the EU previously defined.

### 2.3. Effect of the pH on extraction conditions and stability

**Figure 3.**
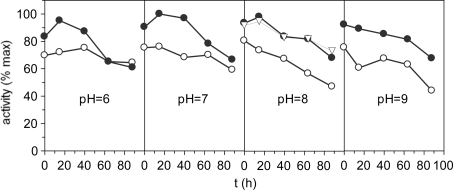
Proteolytic activities as % of the maximum value. •: with 0.2% of Triton X-100; ○: without Triton X-100; ∇: with 0.2% of Triton X-100 and Ca^2+^ 0.02 M. The corresponding confidence intervals of independent experiments are not shown (α = 0.05, n = 3) because they are below 10% of the experimental mean value.

Figure 3 shows effect of Triton X-100, a non-ionic surfactant, at different pH extraction conditions together with stability kinetics. The yields were similar for all pH conditions with a significant increasing (*P* < 0.05) in the range 7–8. In all cases, Triton improves the extractions of enzymatic activity. Nevertheless, the effect of the Ca^+2^ ions concentration tested to pH 8 was not significant regarding non-Ca^+2^ reinforcement. Decreased activities were observed throughout the time for all experimental conditions. Similar results of stability were obtained for proteases isolated from Monterey sardine [33], sardinelle [34] and Japanese sandfish [24].

### 2.4. Joint effect of pH and temperature on proteolytic activity

**Figure 4.**
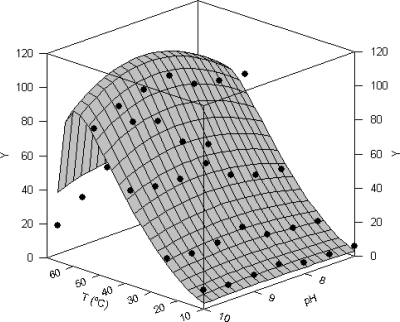
Combined effect of temperature and pH on total proteolytic activity (Y) of rayfish extracts from D section (PRC). Response surface is the fit of the experimental data (•) to the equation (3).

In Figure 4 combined effect of pH and temperature on proteolytic activity of *Procolax* are depicted. The experimental domains were (7–10) for pH and (10–70 °C) for temperature. Response surface was fitted to the modified equation (3) of Rosso described in the Experimental section. The joint optimal pH (*pH_opt_*) and temperature (*T_opt_*) for proteolytic activity were 8.66 ± 0.85 and 59.38 ± 2.23 °C respectively using casein as a substrate. Other interesting parameters obtained from equation (3) were the maximum temperature (*T_max_*) and maximum pH (*pH_max_*) for enzymatic working. These values for *Procolax* were 66.92 ± 0.73 °C and 11.69 ± 2.44, respectively. The fitting of experimental data were statistically satisfactory. The mathematical function was consistent (Fisher’s *F* test; *P* < 0.05) and the parametric estimations were significant (Student’s *t*-test). High coefficient of linear correlation between predicted and experimental values was also obtained (*r* = 0.963).

### 2.5. Partial purification of Procolax

Firstly, precipitation with (NH_4_)_2_SO_4_ was studied. The experimental results are collected in Table 1. The increase of specific activity was low and, taking into account the recoveries of activity obtained, a 60% of saturation is the best option (see column F = C × D). On the other hand, using overcooled ethanol for precipitation no more than 16% of protease and 23% of collagenase was obtained (data not shown). Similar values were released when PEG-4000 was employed as precipitant. However, high total proteolytic activities were recovered with overcooled acetone in high solvent proportions (5 volumes of acetone per residue weight). This last result was very similar to those reported by Michail et al. [35] using cold acetone on partial purification of proteolytic enzymes from trout heads.

Membrane technology working with ultrafiltration-diafiltration scheme was another resource tested in order to collect the enzymatic activity. A molecular cut-off of 675 kD filtrated the most of the protease and collagenase activity. In the retentate, obtained with a concentration factor of 4, only 13.6% (proteases) and 11.3% (collagenases) of the initial activity was recovered. The next step was to process the permeate from 675 kD with a membrane cut-off at 100 kD. In this way, a retentate with a concentration factor of 2, containing 46.7% (proteases) and 47.5% (collagenases) of the initial activity, was performed. Furthermore, specific activities are increased around 15 times and no activity levels are lost in the permeate stream. It is therefore evident that shearing process due to recirculation and pump shear stress lead to enzyme deactivation.

### 2.6. Protocol for Procolax recovery

**Figure 5.**
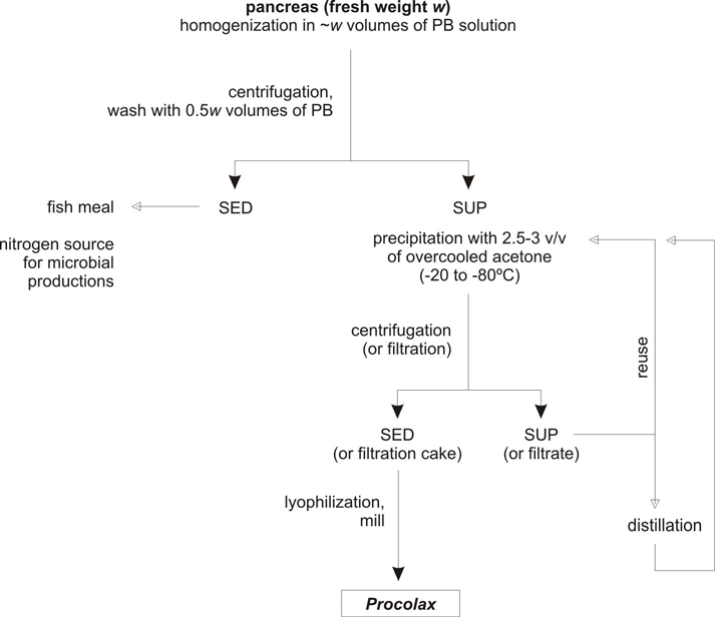
Scheme of *Procolax* preparation from rayfish viscera wastes. SED: sediment, SUP: supernatant. PB solution: phosphate buffer (0.05 M, pH 7.5) with Triton X-100 (0.2%) and KCl (0.08 M).

Bearing in mind the results obtained in previous sections a simple protocol of extraction and purification could be proposed. Figure 5 shows the diagram for the process of *Procolax* recovery. The corresponding stages are as follows (in all cases working at 0–4 °C):

- Initially, a –*w*– weight (in kg) of fresh material (pancreas and duodenum) is homogenized in 1 × *w* volumes (in litres) of phosphate buffer (0.05 M, pH 7.5) with Triton X-100 (0.2%) and KCl (0.08M). Subsequently, the homogenizate is maintained for 1 h with a continuous and soft agitation in order to avoid enzyme deactivation.
- The homogenate is centrifuged (4,000 g for 15 min) to obtain a supernatant with the enzymatic activity and sediment. An additional wash of this sediment with 0.5 × *w* volume of phosphate buffer and subsequently centrifugation in the same conditions produces an increment of 20% in the enzyme recovered. The whole of sediments could be used as source of nitrogen for bio-silage and hyaluronic acid production [32,36] or substrate for fish meal.
- All the supernatants obtained in the previous step are precipitated by means of overcooled acetone addition. Among 2.5 and 3 volumes of acetone are required and slow addition and continuous and soft agitation for 30 min are also necessary.
- The floccules formed when acetone is adding are filtrated via Whatman Nº1 filter. This process of separation is more efficient if floccules are spontaneously sedimented for 30 min and the liquid suspension is drained with a pump.
- The acetone from the filtration cake can be evaporated applying vacuum. Subsequently, the cake is lyophilized and finally milled. The powder obtained is approximately equivalent to the 10% of wet weight from the initial material.
- The hydro-acetone solution from the filtration step could be used again for a new precipitation in combination with pure acetone until 4–6 reuses. From this moment acetone should be rectified by distillation.

The yields of extraction and wash using this protocol are shown in Table 2. Table 3 represents the comparison among enzymatic activities obtained in pancreas and duodenal section without pancreas of purified samples under scheme from Figure 5. Activity was not lost when lyophilisation process was applied to the purified extracts.

## 3. Experimental Section

### 3.1. Waste material from rayfish and reagents

Rayfish (*Raja clavata*) viscera wastes were kindly provided by Dilsea S.L. (the company that manages fish-wastes in the Port of Vigo, NW Spain). For the early studies of proteolytic activity, a division of the gastrointestinal tract in three sections was performed: stomach (S), duodenum including pancreas (D) and terminal intestine (I). Subsequently, these materials were stored in sealed plastic bags at −20 °C until required for enzyme extraction.

In all cases, materials were initially homogenized in phosphate buffer (0.05 M, pH 7.5) with KCl 0.08 M at 4 °C by means of a potter homogenizer (ratio: 20% w/v). The homogenates were centrifuged at 10,000 g for 20 min at 4 °C. The supernatant was washed with the same saline solution and centrifuged again. The supernatants were joined as initial enzyme extract. All reagents used in enzymatic assays were from an analytical grade purchased from Sigma (St. Louis, MO, USA).

### 3.2. Determination of enzymatic activities

#### 3.2.1. Proteases

Protease activity was estimated by the method of Kunitz [37] according to the application of Barker and Worgan [38]. The method is based on the release of tyrosine by the action of protease on casein (protease activity is expressed indirectly in terms of tyrosine concentration). One unit of enzymatic activity (EU) was defined as the amount of enzyme that produced a colorimetric response (λ = 750 nm) equivalent to 1 μmole of tyrosine per minute at pH 7.5 and 37 °C.

#### 3.2.2. Collagenases

The method of determination was based on the leucine liberation from collagen suspension of bovine tendon [39]. It should be pointed out that when this method is applied to extracts with proteases, the produced leucine represents the results of the synergistic effects of proteases and collagenases. Furthermore, as in the case of proteases, protein hydrolysis of the extract could also contribute to the measured leucine. Based on Sigma’s definition, 1 EU is the amount of collagenase (jointly with protease activity) that produces, using ninhidrin reaction (λ = 570 nm), an intensity of colour equivalent to 1 μM (131.2 μg) of leucine with 5 h of incubation, at 37 °C and pH 7.4. For practical reasons, we have used the Sigma definition but with an incubation time of 45 min at 30 °C and pH 7.4.

#### 3.2.3. Proteolytic activities from different intestinal sections

To optimize the quantification of protease activity, homogenates obtained from gastrointestinal sections were assayed at two temperatures (10 and 30 °C), four pH values (2.5, 4, 6 and 8) using 0.1 M citric/phosphate buffer (pH 2.5, 4 and 6) and 0.1 M Tris-HCl buffer (pH 8) as well as two times of incubation (20 and 40 min).

### 3.3. Effect of the pH on extraction conditions and stability

In order to establish the effect of the pH on extraction conditions, the enzyme preparation was incubated at several pH (6, 7, 8 and 9) using different buffers: 0.1 M citric/phosphate (pH 6–7), 0.1 M Tris-HCl (pH 8) and 0.1 M NaOH/glycine (pH 9). In addition, effect of Triton X-100 (0.2%) as detergent and CaCl_2_.2H_2_O (0.02M of Ca^+2^) was also studied. The mixtures were softly shaken and were allowed to rest for 30 min and subsequently centrifuged at 10,000 g for 20 min at 4 °C. The sediments were extracted again in the same conditions and, with the extracts collected, stability was measured for 0, 15, 40, 64 and 88 h at 4 °C. Maximum activity (pH 7 with Triton X-100 and without Ca^+2^ at 15 h) meant 37.3 EU per g of dry weight of duodenum.

### 3.4. Joint effect of pH and temperature on protease activity

Protease activity was measured in a combination of different pH values and several temperatures of incubation under standard assay conditions (pH = 6; 40 °C; 10 min) with casein as a substrate. The enzyme activity was tested at pH (7–10) and temperature (10–66 °C) with 0.1M citric/phosphate buffer (pH 6–7.5), 0.1M Tris-HCl buffer (pH 7.5–9) and 0.1M NaOH/glycine buffer (pH 9–11).

### 3.5. Partial purification of Procolax

An initial solution with 58.54 EU/g of protein was treated with several concentrations of (NH_4_)_2_SO_4_ (20–80%), correcting the pH drop with NH_4_OH when it was necessary. Subsequently, the samples were centrifuged at 10,000 g for 20 min at 4 °C and the resolutions of the sediments were dialyzed at 1 kD cut-off. Similar levels of ethanol overcooled and PEG-4000 were also studied for partial purification of PCR (data not shown). In all these cases, proteolytic activities were only measured.

### 3.6. Analytical methods

Protein concentration was measured by the method of Lowry *et al*. [40] using bovine serum albumin as standard.

### 3.7. Mathematical equation and numerical methods

The joint effect of pH and temperature on the PCR activity was modelled by means of Rosso equation [41,42] modifying microbial growth by enzymatic activity as dependent variable and inserting a new term for pH dependence:

(1) $$f(T)\;=\;\frac{{\left(T-T_{\text{min}}\right)}^{2}\;(T-T_{\text{max}})}{\left(T_{\text{opt}}-T_{\text{min}})[(T_{\text{opt}}-T_{\text{min}})(T-T_{\text{opt}})\;-\;(T_{\text{opt}}-T_{\text{max}})(T_{\text{opt}}+T_{\text{min}}\;-\;2T)\right]}$$

(2) $$f(pH)\;=\;\frac{\left(pH\;-\;{pH}_{\text{min}})\;(pH\;-\;{pH}_{\text{max}}\right)}{\left[(pH\;-\;{pH}_{\text{min}})\;(pH\;-\;{pH}_{\text{max}})\;-\;{\left(pH\;-\;{pH}_{\text{opt}}\right)}^{2}\right]}$$

(3) Y = C · f(T) · f(pH)

where, *Y* is the enzyme activity (%), *C* is the maximum enzyme activity (%), *T* is the temperature (°C), *T_min_* is the temperature below which no activity occurs, *T_max_* is the temperature above which no activity occurs, *T_opt_* is the temperature at which the enzyme activity is optimal, *pH_min_* is the *pH* below which no activity occurs, *pH_max_* is the *pH* above which no activity occurs, *pH_opt_* is the temperature at which the enzyme activity is optimal.

Fitting procedures and parametric estimations calculated from the results were carried out by minimisation of the sum of quadratic differences between observed and model-predicted values, using the non linear least-squares (Levenberg-Marquadt) method provided by DataFit 9.0.59 (Oakdale Engineering, USA). This software was also used to evaluate the significance of the parameters estimated by the adjustment of the experimental values to the proposed mathematical models and the consistency of these equations. On the other hand, the values of the differences among enzymatic activities from different intestinal sections and different pH extraction conditions were subjected to one-way analysis of variance (ANOVA). The means were compared using a contrast test at α = 0.05.

## 4. Conclusions

The present work demonstrates the usefulness of viscera wastes from rayfish to obtain proteolytic and collagenolytic activities in order to upgrade fishing industry by-products. Thus, in addition to the well-known valuable compounds from fish wastes, protease enzymes can also be partially recovered by an easy and sustainable process such as overcooled acetone precipitation. To the best of our knowledge, this work was the first to study enzymic activities from rayfish wastes. Moreover, the activity recovered have a combined optimal of pH and temperature of 8.66 and 59.4 °C, respectively.

## Figures and Tables

**Table 1.** Effect of (NH_4_)_2_SO_4_ on total protein (A), total activity (B), specific activity (C), recovery of activity (D), purification factor (E) and combined response C × D (F).

**(NH_4_)_2_SO_4_ saturation (%)**	**A: Total Protein (g/L)**	**B: Total Activity (EU/mL)**	**C: Specific activity (EU/g protein)**	**D: Recovery (%)**	**E: Purification factor**	**F: C × D**

20	2.78	0.218	78.3	21.0	1.34	1647
40	3.59	0.623	173.6	72.4	2.97	12570
60	4.84	0.804	166.0	86.1	2.84	14299
80	4.49	0.621	138.2	87.0	2.36	12021

**Table 2.** Proportion of activities recovered in the extraction and wash stages.

	**% from the total extract**			**Ratio: first extract/wash**		

	**Protein**	**Proteases**	**Collagenases**	**Protein**	**Proteases**	**Collagenases**

**Extract**	78.1	79.6	83.8	3.6	3.9	5.2
**Wash**	21.9	20.4	16.2	1	1	1

**Table 3.** Comparisons between pancreas and duodenum enzymatic activities in purified samples. Experimental data were expressed as EU per g of dry tissue. Confidence intervals were calculated for α = 0.05 and n = 3.

	**Proteases**	**Collagenases**

**Pancreas**	62.4 ± 5.2	2590 ± 198
**Duodenum without pancreas**	6.5 ± 0.5	660 ± 72
